# The effects of previous educational training on physical activity counselling and exercise prescription practices among physicians across Nova Scotia: a cross-sectional study

**Published:** 2018-11-12

**Authors:** Myles O’Brien, Christopher Shields, Sandra Crowell, Olga Theou, Patrick McGrath, Jonathon Fowles

**Affiliations:** 1 School of Kinesiology, Acadia University, Nova Scotia, Canada; 2 Nova Scotia Health Authority, Nova Scotia, Canada; 3 Divison of Kinesiology, Dalhousie University, Nova Scotia, Canada; 4 Divison of Medicine, Dalhousie University, Nova Scotia, Canada; 5 Divison of Psychiatry, Dalhousie University, Nova Scotia, Canada

## Abstract

**Background:**

Physicians (MDs) report difficulty including physical activity (PA) and exercise (PAE) as part of routine care. MDs who report previous educational training in PAE may prescribe exercise more frequently. We evaluated the effects of previous training on perceptions and practices of PA counselling and exercise prescriptions among MDs in Nova Scotia.

**Methods:**

MDs (n=174) across Nova Scotia completed an online self-reflection survey regarding their current PAE practices. MDs who reported previous training (n=41) were compared to those who reported no training (n=133).

**Results:**

Trained-MDs were 22% more confident performing PA counselling than untrained-MDs (p<0.005). In patient appointments, trained-MDs included PAE more often (51% vs 39%; p=0.03) but trained-MDs and untrained-MDs had similar rates of exercise prescriptions (12%; p>0.05). The most impactful barriers (on a scale of 1 to 4) were lack of time (2.5) and perceived patient interest (2.4), which were unaffected by previous training (p>0.05).

**Conclusion:**

Previous training was associated with a higher confidence to include PAE discussions with patients by MDs in Nova Scotia, but had minimal influence on their many barriers that prevent exercise prescription. Although some training supports MDs inclusion of PAE into their practice, there is a need for greater, more intensive educational training to assist MDs in prescribing exercise.

## Introduction

Physical inactivity has been cited as “the biggest problem of the 21^st^ century” due to its strong association with an increased risk of many chronic diseases such as heart disease, type 2 diabetes, and some cancers.^1^ Despite the undisputed benefits of leading a physically active lifestyle, the vast majority of Canadians do not achieve physical activity guidelines; with only 15% of the population accumulating 150 minutes of moderate-vigorous intensity physical activity per week.^2^ Compared to the rest of Canada, Nova Scotia has higher rates of obesity, cancer, diabetes, high blood pressure, and heart disease all of which may be improved through regular physical activity and exercise.^3-4^

Physicians (MDs) are viewed as an opportune medium of delivering physical activity information and advice due to their frequent interactions with a large proportion of the general public and designation as the preferred source of health-related information.^5^ Previous research demonstrated that patients who receive physician-based physical activity counselling increase their physical activity level.^6-7^ However, MDs experience many barriers to providing physical activity counselling such as lack of time, lack of resource supports and tools, and perceived lack of patient interest.^8-10^ Petrella and colleagues previously observed that only 16% of Canadian family physicians provide patients with written exercise prescriptions.^8^ Furthermore, low self-reported confidence to provide physical activity information, recommend physical activity and exercise (PAE), and appropriately refer patients to qualified exercise professionals are associated with low rates of physician physical activity counselling and exercise prescriptions.^9^

Hoffman and colleagues cited insufficient educational opportunities for primary care providers as a major contributor to the under-prescription of exercise.^11^ This message is consistent among family medicine residents from British Columbia who perceived their exercise prescription training as inadequate (85% of residents) and desired further education as part of their medical training (92% of residents).^12^

Family physicians who receive effective training/education in workshops (employing behaviorist learning theory, competency based curricula, case studies, and practical applications of learning objectives^13^) have higher confidence providing physical activity prescriptions, and in turn, prescribe more frequently.^14,15^ A study of family physicians (n=25) from British Columbia showed the benefits of a physical activity counselling and exercise prescription training workshop on self-reported confidence, knowledge, and rates of physical activity prescription (28% increase in frequency).^16^ On average, people from British Columbia engage in more physical activity compared to Nova Scotians.^17^ In a similar study of MDs across Canada, researchers observed an increase of ~25% in confidence to perform fundamental components of physical activity counselling. This comprehensive workshop increased the proportion of MDs writing exercise prescription increased from 20% to 74% at three-month follow-up.^18^

Canadian-based education/training studies have shown promising results in improving MDs clinical practices, although MDs working in the Atlantic provinces are greatly under-represented. Fundamental principles of medical education stipulate that education should be (1) based on the health needs of the population served, (2) based on the desired needs of the learners, and (3) seamless across the continuum of education, training, and practice.^19^ Considering that rates of chronic disease are elevated in Nova Scotia, there is a pressing need for MDs to assist in the promotion of healthy lifestyles to improve patients’ health and help relieve the economic burden of an over stressed medical system. Whether the role of previous physical activity education or training largely impacts MDs daily clinical practice is unclear, especially in Nova Scotia where the rates of chronic disease are disproportionally high, the geriatric population is growing, and the province is experiencing a doctor shortage.

MDs are in a strong position to promote healthy lifestyle behaviors to the general population but many MDs identify that they have not had the training to deliver such advice and face considerable barriers to do so. The purpose of this study was to evaluate the impact of previous training on perceptions and practices surrounding physical activity counselling and exercise prescription in physicians in Nova Scotia.

## Methods

### Participants

Health care providers across Nova Scotia (n=596) completed an online province-wide survey on PAE practices (FluidSurveys, Ottawa, ON) to identify the state of physical activity counselling and exercise prescription in healthcare generally. The survey was created through an initiative of the Nova Scotia Health Authority Office of Research and Innovation, in collaboration with the provincial health professional associations and prominent provincial disease agencies. Given the purpose of our study was to describe the current PAE practices of MDs in Nova Scotia (n=174), only physician data is presented. The Nova Scotia Health Authority and Doctors Nova Scotia endorsed the survey and distributed it via their employee emails and their respective online newsletters. There are approximately 1352 physicians in Nova Scotia^20^, indicating an estimated response rate of 13% (n=174/1352). To assess the effects of previous educational training, MDs were subsequently grouped into those who self-reported previous physical activity counselling or exercise prescription educational training (n=41) and those who reported no previous training (n=133). Trained physicians were broadly defined as those who reported participating in any of the following PA counselling or exercise prescription related training: medical rounds, webinar, conference presentation, symposium, full-day workshop, half-day workshop, personal exercise experience, academic course, or university degree. The study was approved by the Acadia University and Nova Scotia Health Authority Research Ethics Board and participants provided electronic consent for secondary use of non-identified group data analysis prior to completing the online anonymous survey.

### Providers’ perception and practice questionnaire

We modified previous activity prescription questionnaires,^5,8,9,18,21,22^ (see O’Brien et al. (2017)^9^ and Fowles et al. (2018)^18^) to create the self-reflection questionnaires. We beta-tested the survey and the Exercise is Medicine Nova Scotia Steering Committee edited and approved it. In brief, the self-reflection survey included: demographics, practice history, confidence, and barrier impact. Confidence variables were incorporated because of the known impact on PA counselling self-efficacy.^21^ Questions regarding demographic information, current practice history and barriers were based on published research in diabetes education.^22,23^ Previous training was assessed using a single item to select all the sources of education MDs may have received on PAE. Supporting factors (i.e., ‘facilitators’) that assist physical activity counselling and exercise prescriptions were included from a practical standpoint to describe what physicians are currently doing to incorporate PA into their practice. Practice history and level of confidence were collected using questions covering several domains of practice and confidence, using visual analogue scales from 0-100% (see Appendix A for example). Barrier and facilitator impact was collected using an ordinal scale (1-4) with lower values indicating a barrier or facilitator that has a weak impact on their ability to provide PA counselling or exercise prescription (see Appendix A for example). Conversely, a higher barrier value indicates a very impactful barrier that prevents PAE practices and a higher facilitator values indicates that variable makes it easier to complete PAE practices. Knowledge was assessed using an ordinal scale (i.e., not at all, slightly, moderately, very, extremely). No personal information was required to complete the questionnaire.

### Data and statistical analysis

Descriptive statistics (mean, standard error, proportion, and 95% confidence interval) were calculated on practice, confidence, barrier and facilitator variables using SPSS Statistics Version 23.0(IBM, New York). PA counselling knowledge and exercise prescription knowledge are presented as proportions of very knowledgeable and extremely knowledgeable (%). As previously done in the literature,^9,18^ a self-efficacy composite score was calculated as the sum of all confidence variables, excluding providers’ confidence in patients to follow through on recommendations, as this is confidence in the patient, rather than confidence in themselves. The greatest level of PAE education/training are presented for the trained MDs (i.e., university degree in exercise science provides more education/training than a half-day workshop). A one-way MANOVA controlling for training status, compared PA counselling and exercise prescription practices between trained and untrained MDs. We used Bonferroni *post-hoc* testing to determine within group effects for statistically significant MANOVAs. We also performed *post-hoc* independent sample t-tests, with Bonferroni-correction on confidence, barrier impact and facilitator impact variables due to the difference in the number of MDs who responded to each question and the distinct difference between variables. We set statistical significance at p<0.05.

## Results

### Demographics

The trained group was comprised of those whose highest level of education/training was medical rounds (n=10), took a half-day workshop (n=9), took an academic course (n=7), have a university degree in exercise science (n=6), watched a webinar (n=4), took a full-day workshop (n=3), or attended a symposium (n=2). Some of the trained MDs whose highest level of education/training was a workshop, academic course, or university degree also selected personal exercise experience (n=10), medical rounds (n=8), or webinar (n=5) for a total of 64 responses from the 41 participants. The trained MD group was comprised of mostly female respondents (73.2%, n=30) while the untrained MD group was made up of a similar number of males (51.9%, n=69) and females (48.1%, n=64). Both groups were predominantly Caucasian (trained MDs=90.2%, untrained MDs=85.0%), and of similar age (trained MDs=49±2 years, untrained MDs=51±1 years) and years spent practicing as a physician (trained MDs=20±2 years, untrained MDs=22±1 years). The demographics of participants are presented in Table 1. The majority of respondents saw more than 15 patients per day, spent less than 20 minutes per patient, and worked in a general practice (60%).

**Table 1:** Participant demographics

Demographics	Trained MDs^*^ (n = 41)	Untrained MDs (n = 133)
Age (years)	49 ± 2 [46-52]	51 ± 1 [49-52]
Gender (% Female)	73.2	48.1
Ethnicity (% Caucasian)	90.2	85
Years of Practice	20 ± 2 [16-24]	22 ± 1 [20-24]
> 15 Patients/Day (%)	76.9	59.1
< 20 min/patient (%)	68.3	56.8

* Training status was self-reported; data presented as mean±SE [95% CI] or proportion (%)

### Physical activity counselling and exercise prescription knowledge

A greater proportion of trained MDs reported, with respect to their physical activity counselling knowledge, that they were very knowledgeable or extremely knowledgeable (43.9%, n=18/41) compared to untrained MDs (16.8%, n=22/131). MDs with previous training also reported higher rates of being very or extremely knowledgeable, regarding exercise prescription (34.1%, n=14/41) compared to untrained MDs (10.6%; n=14/132).

### Physical activity counselling and exercise prescription confidence

As shown in Table 2, the self-efficacy composite score (i.e., the sum of provider confidence variables) was 21.8% higher (p=0.004) in the trained MD group compared to the untrained MDs. Trained MDs were more confident (percent difference [%]; p-value) to: provide PAE information (15.4%; p=0.03), answer patients PAE questions (16.9%; p=0.02), help patients maintain PAE (28.3%; p=0.02), assess safety prior to PAE (26.0%; p=0.006), provide PAE advice for those with special considerations (25.6%; p=0.02), and refer to qualified exercise professionals (33.4%; p=0.004). Furthermore, MDs reported low confidence in their patients following through on PAE recommendations, independent of training status (see Table 2).

**Table 2:** Confidence of trained MDs and untrained MDs to provide components of physical activity counselling and exercise prescription

Variables	Trained MDs	Untrained MDs
Provide PAE Information^a^	76.8 ± 4.0^*^ [69-85]	65.0 ± 2.7 [60-70]
Assess PAE Readiness^a^	60.7 ± 4.8 [51-70]	49.8 ± 2.8 [44-55]
Answer Patients PAE Questions^b^	71.4 ± 4.1^*^ [63-80]	59.3 ± 2.6 [54-64]
Help Patients Maintain PAE^a^	38.9 ± 3.7^*^ [32-46]	27.9 ± 2.2 [24-32]
Assess Patient Safety Prior to PAE^a^	62.2 ± 4.8^*^ [53-72]	46.0 ± 2.8 [40-52]
Provide PAE Advice to Patients with Special Considerations (e.g., CVD risk, T2DM)^c^	56.2 ± 4.7^*^ [47-65]	41.8 ± 3.0 [36-48]
Make Appropriate PAE Referrals to Qualified Exercise Professionals^d^	53.6 ± 5.5^**^ [43-64]	35.7 ± 3.0 [30-42]
In Patients to Follow Through on PAE Recommendations^c^	27.4 ± 2.9 [22-33]	31.2 ± 2.2 [27-36]
Self-Efficacy Composite Score^e^	387.8 ± 23.4^**^ [342-434]	295.0 ± 14.5 [267-323]

* p<0.05

** p<0.005

a (Trained MDs: n=40, Untrained MDs: n=130)

b (Trained MDs: n=40, Untrained MDs: n=131)

c (Trained MDs: n=39, Untrained MDs: n=130)

d (Trained MDs: n=39, Untrained MDs: n=129)

e (Trained MDs: n=38, Untrained MDs: n=124)

NOTE: PAE, physical activity and exercise; CVD, cardiovascular disease; T2DM, Type 2 Diabetes Mellitus; the self-efficacy composite score was calculated as the sum of all confidence variables, excluding providers’ confidence in patients to follow through on recommendations; values are out of 100% using a visual analogue scale; data presented as mean±SE [95% CI].

### Physical activity counselling and exercise prescription practices

**Figure 1:**
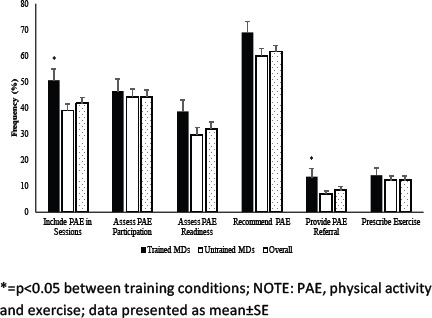
Physician Physical Activity Counselling and Exercise Prescription Practices

Overall, MDs provided written exercise prescriptions in 12.3% of patient sessions, as shown in Figure 1. Those who reported previous training included PAE in more patient sessions than those who did not F(1, 164)=4.72, Wilks=0.236 (50.5±4.3% vs. 38.9±2.6%; p=0.03), as well as provided more PAE referrals to qualified exercise professionals F(1, 164)=4.68; Wilks=0.236, (13.4±3.4% vs. 6.8±1.3%; p=0.03).

### Physical activity counselling and exercise prescription barriers

The highest rated barriers among trained MDs and untrained MDs were lack of time and lack of patient interest (see Table 3). Furthermore, both lack of guidance/resources and lack of appropriate billing structure had an average barrier impact greater than 2.00 out of 4.00. Untrained MDs perceived lack of evidence (p=0.009) and personal knowledge (p=0.001) as significantly stronger barriers to providing physical activity counselling and exercise prescription than did trained MDs, as shown in Table 3.

**Table 3:** Trained MDs and untrained MDs perceived impact of barriers that prevent physical activity counselling and exercise prescription

Variables	Trained MDs	Untrained MDs
Lack of Time^a^	2.49 ± 0.13 [2.24-2.74]	2.45 ± 0.08 [2.30-2.60]
Lack of Exercise Education^b^	1.75 ± 0.18 [1.39-2.11]	2.07 ± 0.09 [1.90-2.24]
Lack of Guidance/Resources^c^	2.06 ± 0.11 [1.83-2.29]	2.29 ± 0.08 [2.13-2.45]
Lack of Evidence^d^	1.03 ± 0.03^*^ [0.97-1.09]	1.28 ± 0.05 [1.17-1.39]
Billing Structure^e^	2.24 ± 0.20 [1.84-2.64]	1.91 ± 0.10 [1.71-2.11]
Personal Knowledge^f^	1.40 ± 0.09^**^ [1.22-1.58]	1.86 ± 0.07 [1.72-2.00]
Patient Interest^g^	2.39 ± 0.12 [2.15-2.63]	2.39 ± 0.08 [2.24-2.54]
Other Changes More Important^g^	1.56 ± 0.10 [1.36-1.76]	1.72 ± 0.06 [1.60-1.84]
Patients Prefer Medication Management^h^	1.97 ± 0.13 [1.72-2.22]	1.91 ± 0.08 [1.76-2.06]

* p<0.05

** p<0.005

a (Trained MDs: n=35, Untrained MDs: n=116)

b (Trained MDs: n=28, Untrained MDs: n=111)

c (Trained MDs: n=35, Untrained MDs: n=107)

d (Trained MDs: n=34, Untrained MDs: n=97)

e (Trained MDs: n=33, Untrained MDs: n=96)

f (Trained MDs: n=35, Untrained MDs: n=108)

g (Trained MDs: n=36, Untrained MDs: n=114)

h (Trained MDs: n=36, Untrained MDs: n=107)

Data presented as mean±SE [95% CI]; Barrier Impact was assessed with an ordinal scale from 1 to 4 with 4 indicating that the barrier completely prevents physicians from providing physical activity and exercise counselling.

### Physical activity counselling and exercise prescription facilitators

MDs rated patient expectations and patient readiness as the most helpful facilitators. Patient expectations and personal comfort/knowledge were significantly higher in the trained MD group compared to the untrained group (see Table 4; p=0.008 and p=0.02, respectively).

**Table 4:** The impact of facilitators in helping physicians perform physical activity counselling and exercise prescription

Variables	Trained MDs	Untrained MDs
Flexibility in booking / scheduling with patients^a^	1.93 ± 0.16 [1.61-2.25]	2.07 ± 0.11 [1.86-2.28]
Support of Practice Group/ Management / Manager / Organization^b^	2.21 ± 0.21 [1.80-2.62]	2.17 ± 0.14 [1.89-2.45]
Readily available resource supports and tools (i.e. pamphlets, websites)^c^	2.44 ± 0.13 [2.19-2.69]	2.43 ± 0.09 [2.26-2.60]
Availability of continuing education opportunities^d^	2.32 ± 0.15 [2.03-2.61]	2.17 ± 0.10 [1.98-2.36]
Patient expectation / interest in physical activity^e^	3.34 ± 0.11^*^ [3.13-3.55]	2.91 ± 0.08 [2.74-3.08]
Personal comfort and confidence in the subject area^f^	3.12 ± 0.09^*^ [2.94-3.30]	2.75 ± 0.09 [2.58-2.92]
Qualified exercise professionals available to refer to as needed^g^	3.10 ± 0.19 [2.73-3.47]	2.73 ± 0.09 [2.55-2.91]
Administrative assists (such as EMR integration of PA, or billing codes)^h^	2.12 ± 0.19 [1.75-2.49]	2.05 ± 0.12 [1.82-2.28]
Patient having greater readiness to do physical activity/exercise^i^	3.29 ± 0.13 [3.04-3.54]	3.15 ± 0.08 [2.99-3.31]
Having physical activity and exercise programs in the community to refer to^j^	3.18 ± 0.13 [2.92-3.44]	3.09 ± 0.08 [2.94-3.24]
Having exercise facilities in the community^f^	3.09 ± 0.14 [2.81-3.37]	3.07 ± 0.07 [2.93-3.21]

* p<0.05

** p<0.005; electronic medical record, EMR; physical activity, PA

a (Trained MDs: n=29, Untrained MDs: n=73)

b (Trained MDs: n=19, Untrained MDs: n=53)

c (Trained MDs: n=32, Untrained MDs: n=84)

d (Trained MDs: n=31, Untrained MDs: n=84)

e (Trained MDs: n=35, Untrained MDs: n=105)

f (Trained MDs: n=34, Untrained MDs: n=104)

g (Trained MDs: n=30, Untrained MDs: n=93)

h (Trained MDs: n=26, Untrained MDs: n=76)

i (Trained MDs: n=35, Untrained MDs: n=105)

j (Trained MDs: n=34, Untrained MDs: n=105)

Data presented as mean±SE

NOTE: Facilitator Impact was assessed with an ordinal scale from 1 to 4 with 4 indicating that the facilitator makes it very easy to provide physical activity and exercise counselling.

## Discussion

The purpose of this study was to evaluate the impact of previous training on perceptions and practices surrounding physical activity counselling and exercise prescription in physicians in Nova Scotia. The overall results suggest that PAE education/training is associated with increased physical activity counselling confidence but not with physician perceived barriers to physical activity counselling and prescription. Additionally, previous training was associated with higher rates of some PAE practices such as providing PAE information and referring to qualified exercise professionals among physicians compared to those reporting no-training. However, training status did not influence provider’s self-reported rates of assessing patients’ PAE level or readiness, recommending PAE, or prescribing exercise. Therefore, medical education and supports for physical activity counseling and exercise prescription in practice should be evaluated to determine how best to influence physical activity prescription rates to patients.

Physicians from Nova Scotia under-prescribe exercise, with providers reporting written exercise prescriptions in only 12% of patient sessions. This is consistent, albeit slightly lower than physicians across Canada, who report prescribing exercise in 16% of patient sessions.^8^ Such infrequent rates are likely attributed to the lack of exercise prescription knowledge, with most physicians self-reporting their exercise prescription knowledge as moderate or lower. The lack of exercise prescription frequency and knowledge becomes a greater issue when factoring in the low frequency of PAE referrals to qualified exercise professionals. Even among physicians who reported previous training, a referral to a qualified exercise professional were included in only 13% of patient sessions, which is further decreased in untrained MDs (7%). The low overall rate of referral aligning with the exercise prescription rate may indicate that referrals are likely well-done when made or may indicate a low availability of programs and professionals to refer to. The lower rate in untrained MDs, likely indicates that knowledge and confidence is also a factor determining which clinical situations may deserve a referral and who can receive referrals for those with chronic disease (i.e., knowledge of appropriate qualifications of exercise professionals), as these domains of confidence are also lower in untrained MDs. This study demonstrates the need for increasing the awareness of, use and collaboration of qualified exercise professionals in the prevention and treatment of chronic disease.

Physicians’ overall self-efficacy composite score was calculated as a broad index of MDs confidence to perform physical activity counselling and exercise prescription and was 22% higher in the trained group compared to the untrained group. Compared to untrained counterparts, trained physicians had 15-30% more confidence performing fundamental components of physical activity and exercise counselling behaviours. This self-efficacy composite score difference is comparable to that observed in pre-post studies in which physicians participated in an exercise prescription training workshop, which observe an absolute increase of 13% in British Columbia physicians at one-month follow-up^16^ and an absolute increase of 40% in physicians across Canada at three-month follow-up.^18^ Petrella and Wight (2000) previously observed that 70% of Canadian physicians claimed to include exercise counselling, but only 67% felt confidence prescribing exercise and the vast majority (94%) were interested in improving their exercise prescription skills.^24^ Based on our findings, it appears that almost two-decades later a similar situation of moderate confidence and an inconsistent application of PAE in practice still exists among physicians, despite a strong interest in improving their ability to provide lifestyle counselling in practice.

The primary barriers indicated by physicians that prevent exercise prescription in the present study were all an absence of factors: time, patient interest, appropriate billing structure, and useful resources to support physicians and patients. This observation is consistent with previous research.^9,14,21^ The vast majority of physicians’ barrier impact ratings (7/9 barriers) were not statistically different (p>0.05) between training status’, with the exception of personal knowledge and lack of evidence for exercise effectiveness. It appears that although trained MDs perceive knowledge and the evidence for exercise as less of a barrier, MDs’ previous training may be inadequate for reducing frequently cited barriers most of which are systemic or environmental. This is further supported by the lack of difference between frequency of assessing PAE participation, PAE readiness, and PAE recommendations between trained MDs and untrained MDs. These findings are in agreement with previous literature reporting that 85% of medical students perceived their physical activity counselling and exercise prescription as insufficient, which may be at least in part, responsible for the infrequent rates of PA counselling and exercise prescription in patient-provider sessions among Canadian physicians.^9,12^ This research demonstrates a need for a more intensive and standardized PA counselling and exercise prescription training across the medical spectrum as it can lead to increased PA by patients and improved body composition, fitness and clinical outcomes.^6,25^ In our sample, only 23% reported any previous training on PAE, and of those, many reported single large group sessions. Specifically, the findings of this study suggest that MDs need more fundamental education on this topic grounded in medical education theory, focusing on time-effective components of PA counselling such as the use of behavioural monitoring with a PA vital sign, motivational interviewing to reframe patient barriers and elicit readiness, and include planning behaviour and goal-setting with specific aerobic and resistance exercise prescription [more practical steps for the inclusion of PAE in primary care provided by Khan et al.]^26^ Educational workshops engaging attendees in these evidence-based strategies have shown great success thus far to reduce barriers and improve prescription practices,^16,18^ but the reach of these training interventions is limited. As such, more bottom-up (current physicians seeking out continuing education) and top-down approaches (i.e., training in medical school) are needed to facilitate and promote the inclusion of PAE as a part of everyday clinical practice. Moreover, while training may modify some variables (i.e., perception of patient interest), it may not change others such as billing structure which requires “up-stream” actions from policy makers and health care decision makers.

Interestingly, the most commonly cited facilitators involve providers’ perception of their patients, including patient expectation or interest in physical activity and readiness to engage in PAE. Given physicians’ reported low confidence in their patients to follow through on PA recommendations, and physicians’ perception of patient interest as a barrier, physicians may unintentionally communicate low expectations of patients’ interest and motivation to avoid difficult discussions about physical activity. Considering the proportion of Canadians who are insufficiently active,^2^ and the physiological benefits of becoming active when previously inactive,^27^ there is a growing imperative for physicians in Nova Scotia to discuss PAE with inactive patients.

### Limitations

Like other cross-sectional studies, group differences are inherently weaker than in intervention based studies; it cannot be confirmed whether providers’ behaviours were solely attributed to previous training. Future research should evaluate the effects of a training intervention on Nova Scotian physicians’ practices, confidence, and barriers. Also, there is a need to investigate the implications of physicians’ providing more PA counselling and exercise prescription, on the actual PA levels and behaviors of their patients.

The type, frequency, duration, and timeline of previous physicians training reported varied, however, the limited sample size did not allow for evaluation of the dose-response relationship of PA counselling and exercise prescription training. The somewhat low response rate (13%) is likely due to busy schedules, the length of the survey (e.g., 15 minutes) and the general invitation sent through the provincial association and health authority emails and newsletters, versus a direct email or in-person solicitation. The individuals who completed the survey did so out of their own interest which may have attracted individuals who already practice physical activity counselling and/or exercise prescription, or at least perceive it as beneficial.

Providers may have responded in what they perceive to be a socially desirable way; reporting higher rates, knowledge, and confidence of physical activity counselling and exercise prescription. Since provider’s self-reported rates of physical activity content in sessions are generally higher than patient reported data and direct observation, they may have over-reported some of their responses in this current study. Given these limitations it is possible, perhaps likely, that the rate of prescribing PA is actually lower than our data indicate.

### Conclusion

Our findings show that physicians with previous training have slightly higher confidence although all physicians experience many barriers that prevent PA counselling and exercise prescription. The current training MDs receive appears to have minimal impact on the environmental and systemic barriers they experience and their inclusion of PAE assessment and prescription as part of their routine clinical practice. Although some training supports MDs’ inclusion of PAE into their practice, there is a need for wide-scale efforts to help MDs meet a higher standard of counselling most patients on physical activity and providing more patients with written exercise prescriptions.

## Appendix A

Screenshot of Example Questions:
